# Exosome and miRNA Content Engagement in the Physical Exercise Response: What Is Known to Date in Atheltic Horses?

**DOI:** 10.3390/ijms27010520

**Published:** 2026-01-04

**Authors:** Giulia Sisia, Elisabetta Giudice, Alessandro Attanzio, Marilena Briglia, Giuseppe Piccione, Caterina Trunfio, Francesca Arfuso

**Affiliations:** 1 Department of Veterinary Sciences, University of Messina, Polo University Annunziata, 98168 Messina, Italy; giuliasisia98@icloud.com (G.S.); egiudice@unime.it (E.G.); gpiccione@unime.it (G.P.); farfuso@unime.it (F.A.); 2 Department of Biological, Chemical and Pharmaceutical Sciences and Technologies, University of Palermo, Via Archirafi 28, 90123 Palermo, Italy; 3 Department of Medicine and Surgery “Kore”, University of Enna, 94100 Enna, Italy; marilena.briglia@unikore.it; 4 Via Sant’Elia, Cardeto, 89060 Reggio Calabria, Italy; catet22@gmail.com

**Keywords:** horses, miRNAs, physical exercise, bioactive molecules, extracellular vesicles, training

## Abstract

To date, there is extensive scientific evidence affirming that physical exercise plays a fundamental role in both the prevention and treatment of various pathological conditions in humans as well as in animals. It is understood that the advantages of movement and exercise have a multifactorial origin and they depend on a category of bioactive molecules vehicolated by extracellular microvesicles known as exosomes. The exosomes act as potential delivery systems for messages within the organism. These findings have drawn significant attention, leading researchers to further investigate the role of exosomes, delving into the study of microRNAs (miRNAs). In particular, these molecules are found inside exosomes and play a key role in cellular communication, with an impact on numerous physiological functions of the organism. It has been suggested that during physical exercise, the expression levels of miRNAs increase in parallel with those of exosomes, and their release enables intercellular communication in multicellular organisms, thereby regulating both cell growth and division. Studies have not only been carried out in humans, but also in laboratory animals and in mammals following exercise. Specifically, a change in exosome expression has been found in athletic horses following physical exercise. The aim of the current review was to highlight what is known about the role played by exosomes and miRNAs during physical exercise in equine species by considering, on a broad scale, the published data on this topic, including comparative data from humans and rodent models.

## 1. Introduction

Physical exercise induces a physiological response in mammals that expands through various adaptations, involving the modulation of multiple energy pathways activated during exercise [1,2]. Physical exercise represents a stressor-inducing systemic response, which involves the hypothalamic–pituitary–adrenal (HPA) axis, the autonomic nervous system, and the immune system [3,4,5,6]. During exercise, horses experience changes in the main regulatory systems that are obviously reversible and restore balance once the exercise ends. However, if the organism is unable to re-establish homeostasis, there could be negative consequences on the animal’s health, welfare, and even on its performance in competition [3,4]. Therefore, the impact of physical exercise on stress and its possible repercussions on health encourage scientific studies to further investigate the physiological and para-physiological mechanisms involved in animal stress [5,6]. In this respect, horses provide an excellent comparative model for humans, displaying similar hematologic and immune adaptations during and after exertion.

The first event in the stress response takes place in the brain: signals originate from it and lead to the release of hormones into the bloodstream, which are then regulated by feedback circuits that can reduce their production. Hormone secretion in response to stress is basically considered an adaptive physiological response, since it allows the organism to cope with temporary conditions and to adapt to new circumstances. It has been shown that following acute stress, the first event is the production of adrenocorticotropic hormone (ACTH) by the pituitary gland, which acts on the adrenal glands to increase cortisol release. It is well known that the release of glucocorticoids and catecholamines after acute stress modulates inflammation to preserve the organism.

Physical exercise represents a moment in which cytokine production is upregulated; cortisol, through feedback, tends to reduce further cytokine release during effort and plays a crucial role in restoring the homeostatic balance of the inflammatory response to exercise, thus preventing the potential onset of pathological conditions [1]. In light of these findings, there is a relationship between serum cortisol concentration, interleukin-1 receptor antagonist (IL-1Ra) levels, and the number of the white blood cells in horses engaged in an official 1300-m race [1]. The horse, being a species capable of training at high intensities, could be considered a model organism for studying adaptive responses to physical exercise. There is evidence that gene polymorphism may be associated with muscle fiber composition and athletic status, as well as the aerobic processes. It has been demonstrated that the AGTR2 gene C allele is associated with an increased proportion of slow-twitch muscle fibers, endurance athlete status, and aerobic performance, whereas the A allele is associated with a higher percentage of fast-twitch fibers and power-oriented disciplines [7]. Moreover, it has been shown that increased SOD2 gene expression is accompanied by decreased lipid peroxidation and increased mitochondrial biogenesis. This indicates that SOD2 mRNA levels in the blood might be useful as a phenotypic biomarker of the muscle-based health-promotion mechanism induced by exercise [8].

**Figure 1:**
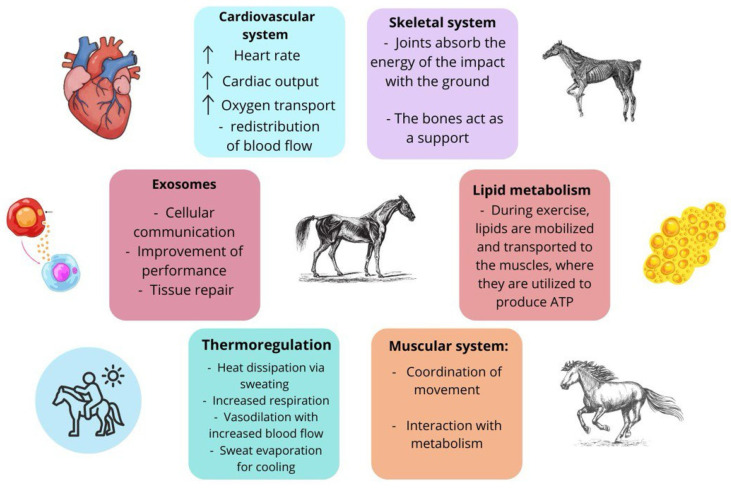
Representation of physiological responses (i.e., cardiovascular, musculoskeletal, metabolic, and thermoregulatory systems, as well as exosome involvement) for athletic horses when exercising.

To date, substantial scientific evidence validates that physical exercise plays a significant preventive role in the treatment of various pathological conditions. During exercise, in fact, positive effects at different levels of the organism have been highlighted (Figure 1). These benefits are multifactorial and depend on a specific category of bioactive molecules, also known as exercise factors [9,10]. Among these molecules, the extracellular vesicles (EVs), including microvesicles (MVs) and exosomes or exosome-like vesicles (ELVs), have been shown to be implicated in the physiological adaptation of the organism to exercise [11,12]. EVs are considered potential mechanisms for the delivery of biological messages within the organism. They are not only involved in cell-to-cell communication, but some evidence also suggests their correlation with, and involvement in, the adaptive response to physical exercise, which is considered highly important [11,12,13].

Several authors have shown that EVs are released together with exerkines, which are also referred to as exercise factors [12]. Therefore, physical exercise promotes the release of EVs, which enables intercellular communication in multicellular organisms, ensuring the development and organization of tissues [13]. EVs, characterized internally by microRNA (miRNA) structures, can differ in size, function, release mechanism, and composition. Interest in exosomes stems from their importance, as they are able to influence various functions by acting on species-specific targets. They are involved in tissue repair, the elimination of obsolete molecules, and in immune surveillance, acting as antigen-presenting molecules [13,14,15,16,17]. In the equine species, racing can cause physiological changes induced by exercise, causing an alteration in protein expression and the metabolic substances within equine plasma EVs. One study focused on analyzing the metabolic content of EVs before and after races in certain horses, providing valuable insights into the role of exosomes in equine physical activity [18,19]. It was observed that, in the post-race phase, the levels of miRNAs related to the cytoplasm and nucleus in plasma EVs increased, together with those miRNAs associated with the transcription of biological processes [19]. EVs have great potential and have attracted considerable scientific interest. This topic is of fundamental importance, as their potential, and consequently, the study of miRNAs in athletic horses, is unique for future non-invasive diagnostics and for understanding the body’s true health status. Research is setting new goals to better understand their function and their correlation with stress factors. In this regard, genomics, transcriptomics, and metabolomics studies are working to provide new insights into the role of EVs and miRNAs, as well as their role in the physiology of athletic horses. The current review aimed to highlight the existing knowledge on the role of EVs and miRNAs during physical exercise in equine species by broadly considering the published data on this topic. Although the primary focus was on studies carried out on athletic horses, comparative data from humans and rodent models were also considered, positioning the equine model within a cross-species molecular framework.

## 2. Literature Search Strategy

To find publications related to the topic, we used the PubMed search engine, searching up to 7 November 2025, using the following terms: “extracellular vesicles in horse” (186 results found), “exercise extracellular vesicles in horse” (4 results found), “miRNAs in horse” (212 results found), “miRNAs in exercised horse” (29 results found), “extracellular vesicles in athletic horse” (8 results found), miRNAs in exercised human (917 results found), “exosomes in horse” (83 results found), and “exosomes in exercise horse” (1 result found). In addition, other relevant publications related to the topic were also included in our research. Though the primary focus was on studies carried out on athletic horses, comparative data from humans and rodent models were considered, positioning the equine model within a cross-species molecular framework. Most studies conducted in the pathological field and/or disease areas were excluded. Due to the diversity of publications on the topic and the novelty of the field, it was not possible to conduct a systematic review. The terminology used within the current review aligns with the rigorous “Minimal information for studies of extracellular vesicles (MISEV2023)” guidelines [20].

## 3. Cellular Vesicle Biology

Cell communication is the process by which cells within an organism communicate with each other, and this occurs through the release of signals that can be direct such as gap junctions, chemical signals at short range such as paracrine and autocrine signals, or long-range signals such as hormonal or nervous communication [14]. Cell communication, or intercellular communication, is fundamental for maintaining homeostasis within organisms. It coordinates cell growth and differentiation, ensures that cells cooperate efficiently, and allows immune system cells to recognize each other and coordinate defense. Cell communication represents a dynamic process through which the organism performs functions that are necessary for proper cellular functioning. It is well established that, in the case of systemic signaling, communication mediated by the receptor–ligand complex occurs through cell membranes and allows the release of extracellular vesicles (EVs) from secretory cells.

**Figure 2:**
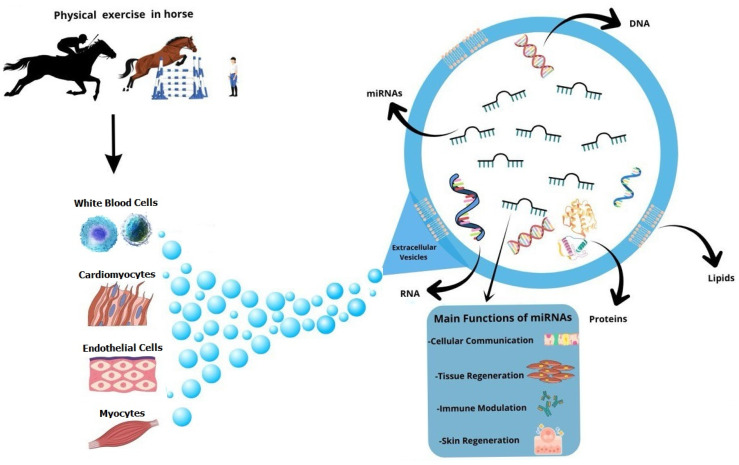
The main roles of EVs described in the horse during physical exercise. Different cell types (endothelial cells, cardiomyocytes, myocytes, and white blood cells) express different types of miRNAs (packaged in EVs), which might influence the function of other cells, promoting cell–cell communication, tissue regeneration, and immune modulation.

Such EVs are involved in various processes within the organism [15,16,17]. Extracellular vesicles are classified into three subgroups: apoptotic bodies, micro-vesicles, and exosomes. The first are formed during programmed cell death and the second derive from budding of the plasma membrane. Exosomes, instead, are vesicles smaller than the previous ones and are characterized by endosomal membranes. They are grouped into multivesicular bodies (MVBs), which are specialized endosomes containing small vesicles called intraluminal vesicles (ILVs), formed through inward budding of the endosomal membrane itself. Previous research has shown that physical activity stimulates the release of exosomes; therefore, the secretion of these extracellular vesicles impacts the body’s adaptation to endurance sports and plays a key role in the repair of damaged muscles [16]. Although the impact of competition on plasma exosome metabolites in equine species is still not well understood, it has been demonstrated that physical exercise could cause modifications in the metabolites of plasma exosomes in horses after physical exercise [18,19] (Figure 2).

### 3.1. Exosomes: Characteristics and Functions

It is now widely recognized that EVs play a crucial role in the physiological response and adaptation of the horse to physical exercise, owing to their ability to repair cell damage and to increase collagen accumulation and migration in horse joint chondrocytes [21], thereby serving as potential biological indicators of physiological recovery capacity [22]. As a matter of fact, exercise stimulates the release of extracellular vesicles into the circulation [14], significantly impacting the physiological functions of exercise [23] by serving as a medium for the body to adapt to endurance sports [24] and playing a role in rebuilding damaged muscles [25]. Among the EVs known, the exosomes, also described as nanovesicles, are small in size, ranging from 30 to 200 nm. They are characterized by marked molecular heterogeneity and are generated through budding. The biogenesis of exosomes represents a protein quality control mechanism, and they perform various activities within the organism, thus promoting proper physiological function [26]. It is important to note that recent studies have highlighted the prominent role of intercellular vesicular trafficking in many aspects of health and disease, primarily in humans. This line of research has also shown that viruses exploit exosome biogenesis pathways both to assemble infectious particles and to establish host permissiveness. Such findings open the possibility of engineering exosomes for pharmacological applications, with potential extensions to veterinary medicine [27]. Moreover, exosomes are surrounded by a phospholipid membrane identical in structure to that of the cell, a feature that allows the application of electroporation—a technique used to load exosomes with exogenous cargo, thereby broadening their applicability in various fields. In genetics, for example, exosomes can be engineered to carry therapeutic payloads, enabling the massive expression of a specific gene or the treatment of a cell line with a specific drug [28]. In regenerative medicine, exosomes are considered ideal candidates within a rapidly advancing scientific landscape [29]. Their qualities—such as stability, bioavailability, and low toxicity—make them highly suitable for use in this field [20,21,22,23,24,25,26,27,28,29,30,31,32,33,34]. Exosomes can serve as transport carriers for MicroRNAs (miRNAs) [35] and they are recognized as useful biomarkers for disease diagnosis [36]. The miRNAs are small non-coding RNA molecules [37] that can regulate gene expression post-transcriptionally and are present in body fluids such as serum, plasma, saliva, and urine [38]. It has been shown that physical exercise and, specifically, the type and intensity of physical exercise, can lead to modification of the plasma levels of miRNAs in humans [39]. Therefore, it has been suggested that miRNAs are a useful tool to differentiate the types of exercise [40] and to assess the physiological response of the organism to exercise [41,42].

### 3.2. Effect of Physical Exercise on Exosome Load

It is now well established and shared by the scientific community that EVs and the miRNAs they contain play a significant role in exercise-induced adaptations both locally, in muscle tissue, and in other target tissues or organs. This is largely attributed to the ability of EVs to efficiently transport molecular cargo, including nucleic acids, and to cross biological barriers such as the blood–brain barrier [43]. These findings suggest that EVs may play a regulatory role together with the endocrine system in the well-orchestrated regulation of physiological adaptations following exercise. To date, several publications have become available in the literature addressing the topic of the influence of physical activity on changes to the EV and miRNA profile in humans [44,45,46,47,48,49,50]. Burke et al. [49] showed that muscle-specific microRNA-1 (miR-1) was transferred to adipose tissue via EVs following an acute bout of resistance exercise. The results gathered in the study highlighted that an overexpression of miR-1 in differentiated human adipocyte-derived stem cells downregulated these miR-1 targets and enhanced catecholamine-induced lipolysis. These data identify a potential EV-mediated mechanism by which skeletal muscle communicates with adipose tissue and modulates lipolysis via miR-1 [49]. Conkright et al. [50] investigated the effect of acute resistance exercise on circulating EV miRNAs and their predicted target pathways in healthy adult men and women (age range, 18–36 yr) performing at least 30 min of physical activity, three times per week, and were free of any injuries that would prohibit participation. Among the results found, the authors emphasized changes in the expression of EV miRNAs targeting growth and metabolism pathways, including p53, IGF-I, STAT3, PPAR, JAK/STAT, growth hormone, WNT/β-catenin, ERK/MAPK, AMPK, mTOR, and PI3K/AKT, and targeting inflammation signaling, including TGF-β, IL-8, IL-7, IL-3, IL-6, IL-2, IL-17, and IL-10 in participants after acute heavy resistance exercise tests. These results seem to suggest that EV cargo, including miRNAs, influences the anabolic environment after resistance exercise. Contrariwise, in agreement with another study [51], changes in myomirs miR-1, miR-133a, miR-133b, miR-206, miR-208a, miR-208b, or miR-499 were found in participants after exercise tests. This finding may be due to the fact that these miRNAs are primarily enriched in muscle [52] and have been previously reported to change skeletal muscle-derived EVs [45,53,54]. However, only a small proportion (1–5%) of circulating EVs originate from skeletal muscle [55], and there is little overlap between circulating EV miRNAs and those derived from muscle [56]. Although the study conducted by Conkright et al. [50] provides novel insights regarding the impact of acute resistance exercise on EV miRNAs and their functional pathways, some limitations should be considered when interpreting the results: (i) the missing assessment of differences in EV profiles according to sex in the context of aerobic exercise, and (ii) the cellular origin of circulating EVs investigated in the study is unknow;, therefore, it was not possible to elucidate the relative contributions of tissues in response to exercise.

Over the last year, the scientific literature has been enriched by studies in the field of sports physiology focusing on exercise-related changes in EVs and the miRNA profile in horses; however, the molecular processes driving the complex post-exercise regulatory network of EV and miRNA cargo are still not fully understood.

It is well established that physical exercise can modify plasma metabolites in horses [57], suggesting that this may cause changes in horse plasma exosome metabolites. However, the impact of competition on horse plasma exosome metabolites is still unclear. A study found that endurance exercise alters the blood metabolomics, transcriptomics, and miRNomics of horses [58]. More recently, Yuan et al., [19], using a multi-omics approach revealed that endurance exercise can alter the metabolomic, transcriptomic, and miRNomic profiles of horse blood, suggesting that there is redundancy and significant changes in the miRNAs, proteins, and metabolites of plasma exosomes. Of note, this study found that the endocytosis process of miRNAs in plasma exosomes after horse racing was enhanced, which may be related to the transport of exosomes mediated by endocytosis [59] and the delivery of miRNAs [60]. Enhancement of the MAPK pathway also supports the speculation that the exercise-induced endocytosis pathway is enhanced [61]. The Kyoto Encyclopedia of Genes and Genomes (KEGG) enrichment analysis showed that the metabolic pathways related to miRNAs in horse plasma exosomes were most enhanced after the competition, suggesting that this is due to the increased cellular metabolic levels caused by exercise and the involvement of exosomal miRNAs in metabolic regulation. As a matter of fact, the Gene Ontology (GO) enrichment analysis showed a significant increase in the miRNAs related to transcriptional regulation in biological processes, as well as an increase in the miRNAs related to the cell membrane composition, cytoplasm, and nucleus, similar to the changes occurring in the miRNAs of skeletal muscle following physical exercise in humans [62]. The results gathered from the study conducted by Yuan et al. [19] showed increased levels of hsa-miR-30a-3p and of hsa-miR-424-3p in plasma exosomes after the competition. The changes in the expression levels of these miRNAs have been demonstrated in tumor tissues and inflammatory conditions (i.e., the expression of hsa-miR-30a-3p is reduced in tumor tissues [59,60] and its downregulation is associated with inflammatory stimuli [63]; the expression of hsa-miR-424-3p is upregulated in tumor tissues [64] and may serve as a prognostic marker [65]). The changes in these two miRNAs found in horses after exercise suggest that they may not only play a role in tumor progression but also a mediating role during exercise, but further research is needed to verify this hypothesis. Moreover, it has been shown that physical exercise inevitably causes metabolic stress, which affects the glycerophospholipids involved in metabolism and in promoting energy supply [19]. The study [19] found that after horses had completed their sporting performance, the levels of glycerophospholipids in plasma exosomes increased significantly. Considering that exosomes, as extracellular vesicles, actively contribute to signal pathway regulation, this study reveals that they also participate in the metabolism of phosphatidylcholine and glycerophospholipids in exosomes. Consequently, glycerophospholipid metabolism is involved in the pathogenesis of rheumatoid arthritis. The KEGG analysis performed in the study showed an increase in the metabolites related to glycerophospholipid metabolism. The same study also demonstrated that higher levels of metabolites such as p-chlorophenylalanine, with its antidepressant functions, and N-acetylcysteine, with its antioxidant functions, improve the physical performance of subjects and actively participate in the physiological processes of exercise, even at high intensity. It has been demonstrated that the levels of these metabolites increase in plasma exosomes after competition, suggesting that horse plasma exosomes are actively involved in transporting the corresponding metabolites to take part in the physical exercise process [19]. The studies undertaken to deepen these dynamics are based on omics technologies, which, in recent years, have undergone considerable development. These technologies, through the use of multi-omics tests, have allowed researchers to understand that the plasma miRNA levels of athletic horses differ and that both the mode of exercise and the degree of training to which they are subjected—relative to the individual capacities of the subjects—inevitably influence disease diagnosis based on miRNAs. Therefore, different levels of competition can modify, and have a direct correlation with, the changes that may develop at the level of plasma exosomes in horses. If exosomes are to be used as biomarkers for post-race diagnosis, it is necessary to understand the specific effects of competition on exosomes to make more accurate assessments.

The correlation between exosomes and physical exercise is now being demonstrated by a growing body of research, to the point that the scientific objective is not only to use them as diagnostic and prognostic biomarkers, but also as tools for monitoring exercise progress, preventing possible injuries, or supporting recovery. They may even provide insights for designing personalized training programs [66]. The evaluation of such studies is clearly applicable not only to humans but also to exercise animals such as horses. Nevertheless, despite these promising objectives that scientific research aspires to achieve, it remains necessary to take into account the limitations and technical challenges currently faced by researchers actively working on these topics [67].

The horse is a high-performing animal with a well-recognized prominent role in athletics, and many authors have investigated the expression of exosomes in this mammalian species in relation to its sports performance. It has been highlighted that one of the responses to aerobic exercise can, in addition to possible skeletal damage in the horse, lead to an increase in the abundance of plasma exosome and miRNA content in this species. The expression of miRNA subgroups is basically tissue-specific, and the extracellular vesicles expressed in a tissue-specific way in the equine species could play a relevant role in cell communication during physical exercise by modulating the expression of miRNAs in the blood and in the skeletal muscle [32]. Therefore, miRNAs are today considered true biomarkers that allow us to distinguish the physiological state in horses. The functionality of exosome expression during the horse’s performance has been deepened. Through KEGG enrichment, a bioinformatic method that identifies the biological pathways and molecular functions associated with a set of genes and proteins, it was found that endocytosis of miRNAs in plasma exosomes in horses increased after races, enhancing the MAPK pathway. This pathway regulates the chain of proteins that enables cellular communication, transmitting signals receptors on the cell surface to DNA in the nucleus. Therefore, KEGG analysis has made it possible to understand that the metabolic pathways related to the mRNAs in the plasma exosomes of horses were significantly improved after competition, suggesting that this is most probably due to an increase in cellular metabolic levels caused by physical exercise and by the consequent involvement of exosomal miRNAs in metabolic regulation [64]. Among the effects of exosome expression during physical exercise, one of the first benefits demonstrated is the effective prevention of cardiovascular diseases. In humans, the molecular mechanism underlying this beneficial effect remains unknown, but previous scientific studies have shown a correlation between the expression of irisin and the expression of exosomes. It is therefore hypothesized that the protective effect of physical exercise against the onset of cardiovascular diseases may be somehow related to the ability of exercise itself to regulate the concentrations of irisin and exosomes in the circulatory system [8,9]. It has thus been demonstrated that during physical exercise, skeletal muscles promote the release of exosomes enriched with irisin, which activates protective networks within the cardiovascular system. The cardiovascular system provides a link between pulmonary ventilation and the cellular utilization of oxygen. Therefore, studies affirm that during physical exercise, it is fundamentally important to ensure oxygen supply to skeletal muscles, which is necessary to sustain ATP production [9,32]. In the case of the equine species, the research indicates that the cardiovascular response of horses to increased oxygen demand during physical exercise contributes to the rise in oxygen consumption that occurs during exercise. Other physiological events also occur during exercise, such as an increase in cardiac output and heart rate, which rises in proportion to the workload until reaching values close to maximum heart rate. Despite variations in cardiac output, increases in blood pressure remain within controlled limits. A higher work rhythm and increased heart rate after training imply what can be defined as an adaptation due to training, which allows for more efficient oxygen supply to the muscles under stress. This adaptation following training is represented both by blood flow and by the arteriovenous oxygen content difference [9,32].

## 4. The Biological Relevance of miRNAs

MicroRNAs (miRNAs) are endogenous non-coding micro-RNAs that actively influence molecular mechanisms through the regulation of post-transcription and mRNA translation. Molecular biology explains that the regulation of gene expression by miRNAs requires the direct interaction of a mature miRNA with the 3′ untranslated region of target mRNAs; any changes in the expression of a miRNA can expose new genes to regulatory functions [66]. In recent years, research and interest in miRNAs has increased significantly because they are involved in numerous processes of development and in the pathogenesis of cancer, as well as in cardiac, immune-related, pulmonary, and other diseases. The miRNAs differ from other types of nucleic acids because they are generally much more stable, even outside the cell, compared to other much longer RNA molecules. This is due to the fact that miRNAs are incorporated into microparticles that protect them. Sometimes, miRNAs can also be found packaged with RNA-binding proteins or lipoprotein complexes, which shield them from harmful enzymes such as RNase. Therefore, many studies affirm that miRNAs can be detected in body fluids during the pathogenesis of various disorders affecting the organism. They can thus be described as promising non-invasive biomarkers of diseases and pathological conditions. It is clear that, compared to humans or mice—frequently used study models—our knowledge of equine genomic regions is considerably more limited. Many research studies reveal that a large number of miRNAs are associated with human diseases; these data demonstrate the potential for research in horses to understand possible pathological conditions through miRNA profiling. Another relevant feature is that miRNAs may also be breed-specific, which represents a key tool since it provides precision and accuracy. Numerous technologies are used in different studies to investigate miRNAs, including sequencing (sRNA-seq) and bioinformatics techniques that provide precise, accurate, and comprehensive profiling of equine miRNAs. A total of 71 sRNA-seq libraries were prepared from six solid tissues, blood, and sera from equine samples, and it was suggested that MiRNAs play a key role in transcriptional and translational regulation in several pathologies. In this study, the expression profiles of miRNAs from the gluteus medius muscle (GM) of healthy horses were compared with those from horses affected by Type 1 Polysaccharide Storage Myopathy (PSSM1). The study highlighted the distribution of RNA fragment sizes, as reported in other equine serum samples, with most reads falling into the RNA region and with a smaller peaks corresponding to the mature miRNA region. Finally, all remaining small RNAs were aligned to the reference genome of *Equus caballus*. Overall, by the end of the study, 91% of reads were mapped; after filtering out multiple mappings, approximately 253,000 unique sequences per library were obtained, of which only 0.39% mapped to the exonic regions of protein-coding genes, 38% to untranslated regions, and 18% to introns. Subsequently, two complementary algorithms—miRDeep2 and miRdentify—were used to identify miRNAs in equine tissues, allowing the discovery of novel candidate miRNAs [67]. A total of eight serum-specific miRNAs were identified: eca-miR-1307, eca-miR-1379, eca-miR-7177b, eca-miR-9021, ecaub_novel-miR-1145, ecaub_novel-miR-262, ecaub_novel-miR-79, and ecaub_novel-miR-932. These were expressed in a tissue-specific manner, although they may also originate from tissues not analyzed in this study. In conclusion, this study enabled the analysis of sRNA-seq data from nine different tissues, providing a global view of both known and novel miRNA tissue distribution. A total of 683 novel equine miRNAs were identified, expressed in seven solid tissues, blood, and serum. Among the results, increased expression of miR-122 and miR-200 was observed in pony serum, along with miRNA target genes involved in energy metabolism. It is hypothesized that the higher expression of eca-miR-483 in ponies compared to horses may be linked to their genetic predisposition to metabolic diseases. The presence of miR-133b and miR-206 was also observed; they were found predominantly in muscle tissue and are actively involved in the differentiation and proliferation of myoblasts [33]. There is increasing evidence that physical exercise stimuli promote the release of exosome-loading miRNAs from skeletal muscle and other tissues into the bloodstream, where circulating miRNAs (ci-miRNAs) can be detected. This strengthens the hypothesis that EVs are important in mediating systemic adaptations following exercise [68]. The ci-miRNAs can be detected in various biological fluids, such as serum, plasma, saliva, sweat, urine, milk, and cerebrospinal fluid [69]. In the bloodstream, ci-miRNAs are transported to the target cells with the aid of EVs (micro-vesicles and exosomes), proteins (Argonaute), or high-density lipoproteins [44]. Most of the studies available in the literature conducted on humans and horses did not examine miRNA profiles isolated specifically from EVs, but rather assessed the total miRNA profile obtained from serum or plasma. As such, the data include miRNAs associated with proteins, lipoproteins, and EVs. Nevertheless, these investigations provide valuable insights into the overall circulating miRNA landscape and may offer indicative information regarding EV-associated miRNAs as well. A study carried out on humans performing resistance exercise showed significant positive changes in ci-miR-133 after acute exercise [70]. Of note, Sawada et al., [71] showed no immediate miRNA profile changes following exercise. Contrariwise, a significant increase in ci-miR-149 was found one day after exercise, whereas the amount of -146 and -221 significantly decreased three days after exercise. In that study, ci-miR-21 correlated with adrenaline and norepinephrine, whereas ci-miR-222 correlated with IGF-1 and testosterone [72], suggesting a relationship with the exercise stress response and muscle gain and strength.

It has been clearly highlighted that using a human plasma panel likely limited the detection of equine-specific miRNAs [18]. Although equine miRNAs have been previously identified, including subsets of tissue-specific miRNAs, scant data on normal patterns of ci-miRNAs as diagnostic tools for different scenarios, including responses to exercise, are currently available in athletic horses [18,19,72,73,74,75,76,77,78,79].

To date, more than 700 equine miRNAs have been identified, with subsets of tissue-specific differentially expressed miRNAs isolated [72], including skeletal muscle and blood [73,74]. Recently, 197 miRNAs were identified in equine skeletal muscle, with 76 found to be muscle-specific [20]. However, to-date there are scant data on ci-MRNA in exercising horses [18,75,76,77,78,79]. Mach et al. [75] identified 167 differentially expressed miRNAs from the blood samples of horses before endurance exercise; however, whole-blood, rather than plasma/serum, was evaluated, making it difficult to determine whether ci-miRNAs were expressed from red blood cells (RBCs) or other tissue sites such as skeletal muscle. There are technical challenges involved in plasma/serum ci-miRNA measurement, such as inaccurately quantifying plasma RNA due to low yields, hemolysis impacting ci-miRNA quantification, and a lack of validated stable reference miRNAs [80,81,82,83]. The possible hemolytic effects on ci-miRNA evaluation is worthy of note, especially in equine species, since hemolysis may occur naturally in horses following moderate-to-intense exercise [84,85]. Therefore, the impact of hemolysis on accurate plasma ci-miRNA quantification represents an important technical challenge and it should be considered when attempting to evaluate equine plasma/serum ci-miRNA expression. In this regard, McGivney et al. [18] carried out a study on Thoroughbred horses in order to identify differentially expressed plasma ci-miRNAs and skeletal muscle miRNAs before and after exercise. In the study [18], the possible influence of hemolysis on plasma ci-miRNA determination in exercising horses was also investigated. McGivney et al. [18] detected 52 ci-miRNAs from the plasma of exercised horses; however, more than 90% of the detected plasma ci-miRNAs correlated with hemolysis, suggesting that an accurate assessment of the changes in ci-miRNA abundance in response to exercise could not be made in horses. It is believed that this technical problem could be overawed through the quantification of exosomal ci-miRNAs (plasma miRNAs packaged in vesicles) rather than assessment of all plasma ci-miRNAs. As a matter of fact, the quantification of exosomal ci-miRNAs would exclude all unpackaged miRNAs that may have leaked into the plasma from hemolyzed erythrocytes. Of note, the researchers detected interesting changes in equine skeletal muscle miRNA abundance following exercise [18]. Two members of the let-7 family of miRNAs (i.e., let-7d-3p and let-7d-5p) and miR-21-5p increased their expression following exercise. The Let-7 family of miRNAs are involved in key processes in energy metabolism during exercise, including the regulation of glucose homeostasis and insulin sensitivity [86]. Mir-21 might play a role in muscle remodeling during exercise training, as it has been shown to be involved in the fibrogenic pathway and in Duchenne muscular dystrophy evolution [87]. Contrariwise, two members of the miR-30 family (i.e., miR-30b-5p and miR-30e-5p) exhibited decreased expression after exercise. In vitro analysis in mice indicated that miR-30 family decreased following injury and increased during myoblast differentiation [88]. These findings suggest that these miRNAs may play an important role in skeletal muscle growth and repair [88] and that the decreased expression in response to exercise may be related to the repair of exercise-induced muscle damage. Overall, the changes in expression of these miRNAs following exercise underpinned the key role played by these molecules in the modulation of gene expression in response to exercise [18].

Kim et al. [76] investigated the relationships of miRNAs from leukocytes with exercise in Warmblood horses. In the analysis of the miRNAs, 229 known miRNAs and 150 novel miRNAs were found to be altered in circulating leukocytes after exercise. Significantly, four known miRNAs and two novel miRNAs had common changes in expression among three horses following one hour of exercise, suggesting these are exercise-specific miRNAs in blood leukocytes. The known and novel miRNAs were eca-miR-144, eca-miR-33a, eca-miR-545, eca-miR-423-5p, novel miR-14-5p, and novel miR-95-3p. In the present study, miR-144 and miR-33a matched the exercise-specific miRNAs in animal studies. The miR-144 expression decreased in this study, and it has been observed to increase in the heart muscles of rats after one hour of swimming [89]. The differences between these two studies, such as the species, the circadian systems characteristics of diurnal and nocturnal animals, the small number of samples and its origin, and the exercise type and intensity, may be the cause of their contrasting gene expression results. Additionally, miR-33a, which was downregulated after one hour of exercise in three Warmblood horses, increased after 30 min of trotting in Thoroughbred horses [74]. This may result from differences in the breed, the type and duration of exercise, and the history and life management previously entrained by exercise or the photoperiod [90]. The functions of the novel miRNAs are presently unreported, which warrants further studies to identify the roles of the miRNAs in exercise. However, the results have been obtained from the integrated analysis of only two Warmblood horses without considering the influence of circadian rhythms associated with daytime scheduled exercise on the altered expression patterns of miRNAs; thus, they need to be interpreted cautiously to avoid over-interpretation.

Another study [76] investigated the serum profile of ci-miRNAs in response to prolonged endurance exercise in samples obtained from competitive horses through massive parallel sequencing. The authors identified a large set of modulated ci-miRNAs and target genes that clustered in protein–protein interaction (PPI) networks, revealing that responses to endurance exercise induce major changes in muscle remodeling, metabolic pathways, and, ultimately, in the immune system and inflammatory response mechanisms. Therefore, modulated ci-miRNAs in endurance athletes can be considered promising and reliable biomarkers of stress and/or training. Major changes were observed in circulating levels of eca-miR206, 133a, 133b, 208b, 499-5p, and 486-3p, which are muscle-specific miRNAs (i.e., myomiRs) derived from muscles and the heart. The expression of miRNAs eca-miR206, 133a, 133b, 208b, and 499-5p showed strong upregulation in horses after exercise, whereas the miR-486 was the most downregulated. The authors stated that the changes in ci-miRNAs occurring in the investigated horses could be the result of active or selective secretion rather than muscle cell damage, as suggested by the increases in ci-miR-1, ci-133a, and ci-206 expression, which are likely correlated with performance parameters and are poorly associated with biochemical markers of cardiac and/or skeletal muscle damage, and by the decrease in the classical myomiR ci-miR-486. Additionally, miR-199 and the miR-99/100 family, which play a role in the maintenance of cardiac homeostasis and in regulating apoptosis in cardiomyocytes through their target, IGF1R [91], were found to be upregulated in horses after exercise. The results showed the activation of immune and inflammatory responses via the modulation of miR-224 and miR-1180 through a key immune systems regulator, transcription factor κB (NF-κB) [92]. Indeed, inflammatory stimuli, such as strenuous exercise, can induce the activation of the innate immune system via miR-224 and the pentraxin 3 (Ptx3) gene, both mediators of inflammation; miR-224 is a transcriptional target of NF-κB, and the Ptx3 promoter contains an NF-κB binding site for its regulation [93]. The upregulation of miR-224 leads to the activation of EGF, transforming growth factor alpha (TGF-a), IGF, and tumor necrosis factor α (TNFα), inducing immune cell proliferation and migration via inflammatory response signals [94]. The downregulation of miR-1180 suggests modulation of immune cell survival and proliferation by directly targeting key inhibitors of the NF-κB signaling pathway and the apoptotic protein Bcl-2 [95].

In the study conducted by Ma et al. [78], the dynamic miRNA expression profile associated with exercise-induced stress in Yili horses during a 5000-m race was characterized, identifying specific miRNAs including miR-1 and miR-486-3p as potential biomarkers for breeding selection. In particular, post-exercise upregulation of miR-1 and miR-143 in Yili horses was observed, and it has been suggested that thes miRNAs may mediate physiological adaptations to exercise, producing positive effects such as enhanced energy supply during racing through the modulation of fatty acid metabolism, improved structural integrity of muscle fibers, better resolution of inflammation and tissue repair, enhanced muscle microcirculation, the protection of vascular endothelium from oxidative damage, and the promotion of osteoblast differentiation. However, in this study, only the top three winning horses (all males and 4 years of age) were investigated. This limited sample size may introduce certain limitations to the accuracy and generalizability of the findings. Moreover, the influence of factors, including age, sex, and breed, warrants further investigation.

Most of the discussed studies assessed the total ci-miRNA profile in plasma or serum, which included the miRNAs not only encapsulated within EVs, but also those bound to proteins or associated with lipoproteins. The miRNAs may enter the circulation through non-specific pathways rather than via targeted cell-to-cell communication; therefore, the resulting data may not accurately reflect the biological processes directly related to exercise-induced adaptations. This could compromise the interpretation and reliability of the conclusions drawn. Therefore, further investigations aiming to elucidate the adaptive mechanisms triggered by physical exercise are necessary and should primarily focus on the miRNAs specifically transported by EVs.

## 5. Possible Role of miRNAs on Equine Asthma and Osteoarthritis Biology: Scientific Evidence

Horses are remarkable athletes whose respiratory systems are supremely capable of delivering high airflow and the oxygen that is necessary for superior athletic performance. Both nature and the environment interfere with this oxygen delivery, resulting in poor performance in the form of both upper and lower airway diseases. Equine asthma (EA) has a broad impact on the sport horse population. EA, as a disease of domestication and exposure to particulate matter, affects all groups of horses, including endurance horses, which do not perform under conditions that produce high pulmonary artery pressures. EA is a disease that causes airflow impairment, which increases in severity with exercise. It has been recognized previously as the second most common cause of poor performance in racing horses [96].

Recent studies state that neutrophilic inflammation is associated with respiratory disease in horses; this inflammation has been linked to airway obstruction in severe equine asthma (SEA). According to this study, it seems that neutrophils play a major role in causing true structural alteration of the airways, and this would occur precisely through the release of exosomes [97,98,99,100,101,102,103,104,105]. Scientific data suggest that neutrophil-derived exosomes promote airway smooth muscle (ASM) cell proliferation, both in humans and in horses. This was considered the hypothesis for identifying neutrophil exosomal microRNAs involved in the regulation of both ASM biology and SEA [57]. The methods used to investigate neutrophil microRNA expression in horses consisted of quantitative PCR, a technique that allows real-time detection or quantification of gene expression. In practice, PCR requires specific reagents depending on its features [104]. In this particular study, it enabled a detailed and quantitative analysis of the expression of selected miRNAs in exosomes. Subsequently, the effect of miR-21 transfection into ASM cells was also evaluated through gene expression analysis and proliferation studies. In this study, whether low levels of exosomal neutrophil-derived microRNA-21 contribute to airway smooth muscle hyperproliferation in horses affected by severe asthma was assesssed and it was revealed that microRNA-21 was downregulated in neutrophil-derived exosomes of SEA horses and attenuated the proliferation of ASM cells that had been stimulated with lipopolysaccharide. Therefore, based on the results obtained, it was possible to conclude that lower levels of miR-21 in neutrophil-derived exosomes may actively contribute both to airway complications in horses already affected by severe asthma and to what is described as bronchial wall thickening in SEA. A further scientific study also revealed that there are molecular pathways responsible for ASM remodeling, but these are still not fully understood [104]. It is already known that miRNAs are key regulators in inflammatory and reparative processes affecting the lungs. For this reason, researchers assume that they may also be involved in ASM remodeling by modulating proliferation [105]. The expression of miRNAs in bronchial smooth muscle was analyzed in asthmatic horses, horses in remission, and healthy controls [104]. The study demonstrated the possible involvement in ASM cell proliferation. The results effectively showed that miR-26a, miR-133, and miR-221 were upregulated in the ASM of asthmatic horses compared to those in remission and in healthy controls. According to the data collected, it seems that miR-221 induced cell hyperproliferation and reduced the expression of genetic markers in ASM cells. The results were also associated with reduced mRNA expression of cell cycle regulatory genes, namely p53, p21, and p27. Thus, the study demonstrated that upregulation of miR-221 in asthmatic airway smooth muscle is involved in ASM cell proliferation through the regulation of cell cycle arrest genes. A more in-depth study on the targeting of miR-221 network genes could represent, in the future, a novel approach for treating ASM remodeling in equine asthma [105].

In Table 1 the main miRNAs with known functions identified in asthmatic and healthy horses have been reported.

Over recent decades, the presence of EVs in the secretome of mesenchymal stem cells (MSCs) has been demonstrated as special mediators of inflammation [106,107,108,109,110]. Of note, the supportive effects of EVs and their miRNA content for the treatment of osteoarthritis has been shown [111,112]. The miRNAs in EVs, both from undifferentiated MSCs, which could be used for the treatment of osteoarthritis, and from chondrogenically differentiated MSCs, as a possible damage model of impaired repair in joints affected by osteoarthritis, have been investigated [105,106]. Moreover, using an equine model, it has been determined how the content of EVs change when they are not kept under comparatively normal (cell culture) conditions, but instead are stimulated in different ways [107]. This involved either a damaging stimulation, such as treatment with interleukin 1β, or pro-cartinogenic stimulation in the form of chondrogenic differentiation of MSCs [110].

The miRNAs were extracted and analyzed from the EVs of equine adipose-derived mesenchymal stem cells. A total of 89 miRNAs, whose expression was significantly altered compared with that of an untreated negative control, were identified. On average, 53 miRNAs were upregulated and 6 miRNAs were downregulated. Among others, the miRNAs eca-miR-101, eca-miR-143, eca-miR-145, eca-miR-146a, eca-miR-27a, eca-miR-29b, eca-miR-93, eca-miR-98, and eca-miR-221 were significantly increased after the stimulations, which, as known ant-inflammatory miRNAs, could be candidates for therapeutic use in the treatment of osteoarthritis [110].

## 6. Discussion

In recent years, research on EVs has been rapidly increasing and developing due to the great interest these molecules have generated thanks to their high potential and the numerous applications they can be subjected to, both in veterinary medicine and beyond. They can, in fact, be used as true methods for monitoring health status, as well as therapeutic vehicles in the field of regenerative medicine.

It has also been established that the nucleic acid and protein content of exosomes can be taken into consideration to anticipate physiological conditions or possible pathologies in athletic horses, with the aim of improving both their health and their performance. This broadens the diagnostic perspectives, making it possible to create strategies that are increasingly timely and that can also positively impact the development of targeted treatment approaches.

According to the currently available scientific literature, it is clear that exosomes and, consequently miRNAs, play a role in the well-orchestrated mechanisms of physiological adaptation to physical exercise, not only in humans but also in equine species. This is not surprising, considering the fascinating, well-defined role of exosomes in intercellular communication. However, there is still much to hypothesize and substantiate with scientific evidence.

The scientific community, particularly those studying physiological responses and/or pathological conditions in horses, should focus their attention on exosomes and their content with the goal of gaining a deeper understanding of their modulation within the organism, so as to expand not only knowledge in the genomic field but also to enhance their role as biomarkers to be used in diagnostic and prognostic contexts in a non-invasive manner.

Due to the variability in exercise types, duration, intensity, and frequency, it is difficult to detach the specific effects of exercise on miRNAs and EVs, impeding consistent and conclusive results. For the investigation of EVs in sport sciences, it would be advisable to follow the current MISEV guidelines [20]. These principles have also been summarized from a physical exercise viewpoint [23].

From the published research papers on humans and horses, it emerged that, following exercise, the EV and miRNA profiles not only contribute to local adaptations at a muscular level, but EV-derived miRNAs may have a positive effect on other tissues, influencing, for instance, lipid metabolism. However, the molecular cascades influenced by EV loads are not fully understood. Therefore, scientific research should be intensified in order to better understand and define the role of EVs and miRNAs in the physiological adaptation of animals to physical exercise, both at the muscular level and at the level of other functional systems of the organism. This could give useful information that would allow trainers to adapt exercises based on an individual’s specific needs, goals, and potential physical limitations in order to optimize the benefits of exercise, as well as performance. It should not be overlooked that research on exosomes holds value and potential, rather, it can be stated that the advances we are witnessing, and will continue to see, in exosome research, together with genetic insights into miRNAs, represent a crucial step that could provide significant support to the diagnostics both in human and veterinary medicine, ultimately ensuring more effective solutions to health-related issues, not only in human medicine but also in the veterinary field.

## 7. Conclusions and Future Perspectives

A greater understanding of the role of exosomes in exercising horses could assist and simplify the diagnostics of these animals, helping to improve their welfare and, consequently, their performance in competition. However, the studies carried out on this topic in athletic horses showed several limitations that should be considered when interpreting the results. As a matter of fact, several papers addressing the effect of exercise on EVs and/or miRNAs in horses lacked evaluation of the influence of factors, including age, sex, and breed, on EV profiles. To date, no studies have been carried out on exosomes and miRNAs comparing the athletic performance or response to exercise among different horse breeds and/or among horses distributed across various continents and climates. The possible impact of these variables on the physiological response of athletic horses to physical exercise, as well as on the exosomes and miRNA involvement, would be worthy of investigation. Moreover, most of the currently available papers on this topic in athletic horses did not clearly show the cellular origin of circulating EVs investigated, making it difficult to elucidate the relative contributions of EVs and/or miRNA related-tissues in response to exercise. Another recurring limitation in currently published manuscripts on the effect of physical exercise on athletic horses is the size of the investigated sample, which is often very small, compromising the accuracy and generalizability of the findings. Many studies focused on the assessment of the total ci-miRNA profile in plasma or serum from exercising mammals, including horses. However, these total ci-miRNAs included the miRNAs not only encapsulated within EVs, but also those bound to proteins or associated with lipoproteins. Considering that miRNAs may enter the circulation through non-specific pathways, the resulting data may not accurately reflect the biological processes directly related to exercise-induced adaptations, compromising the interpretation of the gathered findings. In view of these limitations, and considering the remarkable potential and functional significance of EVs and/or miRNAs in understanding the physiological adaptations of athletes to physical exercise, the researchers working in the field of sports physiology should intensify their studies in this field and primarily focus on miRNAs specifically transported by EVs in order to elucidate the adaptive mechanisms triggered by physical exercise. Further investigations should be carried out to identify the possible influence of breed and age on the exosomes and microRNAs in the body’s response to exercise in sports horses. Finally, the functions of the novel miRNAs, which are presently unreported, require further study to identify their roles in exercise. Last but not least, the influence of circadian rhythms associated with daytime scheduled exercise on the altered expression patterns of miRNAs is worthy of investigation.

## Figures and Tables

**Table 1:** The main miRNAs with known functions identified in equine species.

Equine miRNAs with Known Functions			
miRNA ID	Tissue	Function	Reference
miRNA-21	Myeloid	Hyperproliferation of the smooth muscles of the airways	Vargas et al. [104]
miRNA-26 a	Muscle	Upregulation in the ASM of horses with asthma	Issouf et al. [106]
miRNA-133	Muscle	Upregulation in the ASM of horses with asthma	Issouf et al. [106]
miRNA-221	Muscle	Upregulation in the ASM of horses with asthma-induced cell hyperproliferation and reduced expression of genetic markers in ASM cells	Issouf et al. [106]
miRNA-122	Liver	Regulation of energy metabolism	Pacholewska et al. [33]
miRNA-200	Blood (serum)	Regulation of energy metabolism	Pacholewska et al. [33]
miRNA-133 b	Muscle	Differentation and proliferation of myoblasts	Pacholewska et al. [33]
miRNA-206	Muscle	Differentation and proliferation of myoblasts	Pacholewska et al. [33]

## Data Availability

No new data were created or analyzed in this study. Data sharing is not applicable to this article.

## Abbreviations

The following abbreviations are used in this manuscript:

miRNAs	microRNAs
EVs	extracellular vesicles
ci-miRNA	Circulating miRNA
MISEV	Minimal Information for Studies of Extracellular Vesicles
ACTH	adrenocorticotropic hormone
IL-1Ra	interleukin-1 receptor antagonist
IL-1	interleukin 1
TGF-β	transforming growth factor-beta
IL-8	interleukin 8
IL-7	interleukin 7
IL-3	interleukin 3
IL-6	interleukin 6
IL-2	interleukin 2
IL-17	interleukin 17
IL-10	interleukin 10
MVBs	multivesicular bodies
ILVs	intraluminal vesicles
sRNA-seq	miRNA sequencing
GM	gluteus medius muscle
PSSM1	Type 1 Polysaccharide Storage Myopathy
SEA	severe equine asthma
ASM	airway smooth muscle
